# Spatial Transformation Characteristics and Conflict Measurement of Production-Living-Ecology: Evidence from Urban Agglomeration of China

**DOI:** 10.3390/ijerph19031458

**Published:** 2022-01-27

**Authors:** Yu Chen, Xuyang Su, Xuekai Wang

**Affiliations:** 1 School of Economics and Management, Zhengzhou University of Light Industry, Science Avenue 136, Zhengzhou 450000, China; 2012030@zzuli.edu.cn (Y.C.); suxuyang_123@163.com (X.S.); 2 National Academy of Economic Strategy, Chinese Academy of Social Sciences, No.1 Dongchanghutong, Beijing 100006, China

**Keywords:** production-living-ecology, spatial transformation characteristics, spatial conflicts, urban agglomeration

## Abstract

The land development by human beings has changed from the initial small-scale and low-level transformation to the comprehensive utilization of large-scale and high-intensity implementations. The contradiction between production-living-ecology space (PLES) has become increasingly prominent while drawing land dividends. As one of the important birthplaces of Chinese civilization and the ecological barrier in the northern region, the rapid urbanization and industrialization of the Yellow River Basin (YRB) make the ecological environment very fragile, and the imbalance of land and space development is extremely serious. Therefore, according to the multifunctional characteristics of land use, this paper establishes a classification system of production space (PS), living space (LS) and ecology space (ES), and discusses the spatiotemporal evolution and conflict distribution characteristics of the PLES with the help of the transfer matrix and spatial conflict index (SCI). The results are as follows. In 1990–2020, agricultural production space (APS), grassland ecology space (GES) and other ecology space (OES) yielded the largest proportion of PLES in the YRB. However, compared with 1990, the area of these spatial types decreased in 2020, while the urban living space (ULS) expanded rapidly. The distribution pattern of PLES was generally consistent, and the transformation between PLES in Ningxia, central Inner Mongolia, Loess Plateau and downstream areas was relatively intense. The conflict index of PLES showed an upward trend, but it was generally in a controllable range. The stable and controllable areas were concentrated in the upstream of the urban agglomeration, and the midstream and downstream were basic out of control and seriously out of control, respectively.

## 1. Introduction

Territorial space is the carrier of socioeconomic development, as well as the support of human survival and development, and the evolution of its pattern and function is the result of the continuous interaction between humans and land [1,2]. The imbalance between the supply and demand of land resources is increasingly fierce with the construction of modernization, and limited land resources are reallocated quantitatively and spatially in the game among various interests. This dynamic process is known as land use transformation, which reveals the transformation of land use patterns corresponding to the stage of economic and social development in a certain period driven by various internal and external factors [3,4]. However, a healthy land use system has not only structural integrity, but also functional continuity and additivity [5]. Since the beginning of the new century, China’s rapid population urbanization and land urbanization process have accelerated the demand for land resources [6,7,8], and urban and rural construction land has continuously encroached on agricultural and ecological land, causing increasing land use conflicts in space. In essence, land use conflict is the competition for resource elements in time and space among land-using subjects [9], which is manifested as the uncoordinated development of the human-land relationship. Once the conflict level is intensified or out of control, it will lead to a series of problems such as the waste of spatial resources, destruction of ecosystem, weakening of the stability of the landscape pattern and imbalance of social development [10,11,12,13,14,15].

Since the beginning of the new era, China has attached great importance to land planning to address a series of negative problems caused by the overexploitation of land resources. The report of the 18th National Congress put forward the overall construction goal of “Intensive and efficient in the production space, livable and moderate in the living space, beautiful in the ecological space”, and made optimizing the spatial development pattern of the country the first step in the construction of ecological civilization. In 2017, the National Land Planning Outline was released to make specific arrangements for regional development, the construction of the main functional areas and development goals, and in the report of the 19th National Congress, it was further proposed to carry out the delineation of three control lines, namely the “ecological protection red line, permanent basic agricultural land and urban development boundary”.

The YRB is a typical region of rapid economic and social transmutation [16,17,18]; has the PLES spatial pattern in the YRB changed significantly in recent decades? Will the spatial conflict index increase with socioeconomic development? Therefore, the YRB is used as the research object of PLES transformation characteristics and conflict measurement mainly based on the following considerations: (1) Important strategic positioning: The Outline of the Plan for Ecological Protection and High-Quality Development of the YRB regards this region as an important benchmark for the management of large rivers, an important benchmark for ecological safety, an important test area for high-quality development, and an important carrying area for the preservation, inheritance and promotion of Chinese culture. Research on the spatial pattern of PLES in this region is conducive to practicing the concept of ecological civilization construction and building a new pattern of land space development. (2) The unique geographical location: The YRB straddles three major steps in China, with a variety of terrain and landforms crisscrossing the basin and a weak resources and environment carrying capacity. The study of the spatiotemporal patterns and conflict levels of the PLES is conducive to the implementation of refined management, and to solving the outstanding problems of land grabbing for economic and social development in the YRB. (3) The intricate economic and social environment: After decades of reform and opening up, the level of urbanization and industrialization has improved significantly. However, problems such as uncoordinated urban and rural development, unbalanced regional development, the contradiction between economic growth and ecological protection, etc. are more serious. In addition, the ecological environment is fragile, and water resources are extremely scarce. The interaction and coupling system of land relationships demonstrates strong incoordination. Research on the conflict of PLES in this region is helpful to understand the current situation of land use conflict, optimize land use function and improve land use quality.

**Figure 1:**
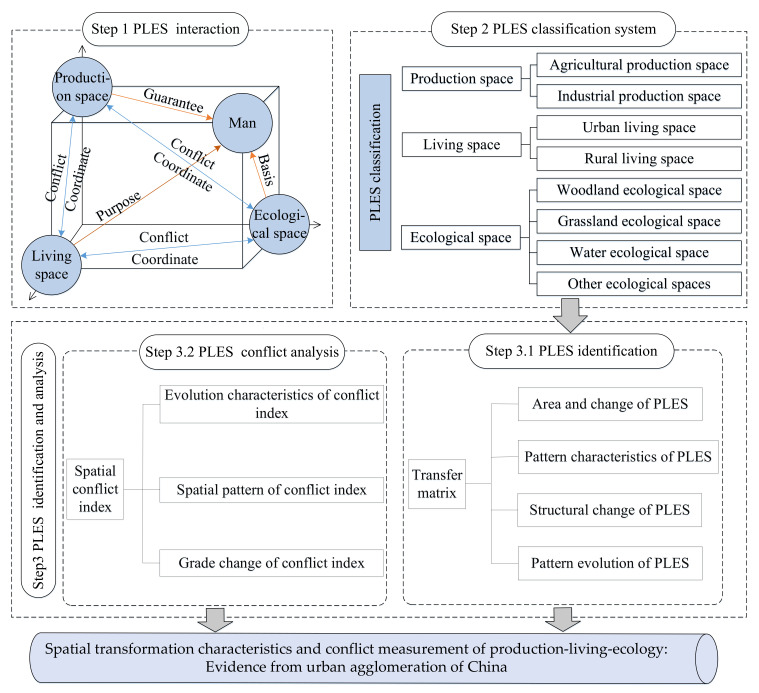
Research framework. Source: The authors.

The structure of this paper is as follows: Section 2 is a literature review on the study of PLES; Section 3 is a presentation of data and methods; Section 4 is an analysis of the evolution, patterns and conflicts of PLES; and Section 5 is a conclusion and suggestions. Abbreviations attached at the end of this paper (Table A1). The research framework is shown in Figure 1.

## 2. Literature Review

In the 1980s, western countries launched a number of spatial planning research programs. By establishing evaluation models and using GIS and other methods, the suitability and mode of land spatial development were evaluated from the perspective of environmental protection [19]. As research progresses, the multifunctionality of land use is further subdivided into economic, social and ecological functions [20,21,22]. The study of PLES originated from agriculture in the Taiwan Province, which was originally aimed at balancing agricultural production and protecting the ecological landscape, but later evolved into the study of dividing the whole national space into production, living and ecology space. At present, from the perspective of land use, ecosystem and landscape value, research on PLES mainly focuses on identification and classification, the analysis of spatiotemporal evolution characteristics and influencing factors, spatial optimization and spatial conflict.

### 2.1. Identification and Classification of the PLES

The quantitative identification of the PLES is mainly based on the measurement of spatial quantity or quality, and the common measurement methods include land use type consolidation and the comprehensive index evaluation. On the one hand, from the existing research results, the method of land use consolidation is the most widely used, and it is based on the functions carried by each land use type to identify the pattern characteristics of the PLES intuitively and quickly [23,24]. However, scholars differ in their understanding of land-bearing functions, which are mainly divided into single spatial division and composite spatial division. Among them, scholars who hold a single spatial view believe that each land use type only considers its dominant function, and the division results in three spatial categories including production, living and ecology space, with no overlapping areas between the spaces [9,13,25]. Some scholars also believe that land can carry multiple functions at the same time, in addition to the single function of production, living and ecological, but also, has three spatial types of composite functions [26], for example, cultivated lands have both production and ecological functions, so the land is divided into production-ecological, living-production, etc., which is a composite spatial division. On the other hand, the method of the evaluation and measurement of the index system is mainly based on the economic, social and environmental factors related to production, living and ecological, and the construction of a comprehensive evaluation index system, such as the general evaluation index [27], coupling and coordination evaluation index [28], spatial suitability evaluation index [29] and resource and environmental carrying capacity evaluation index [30], etc., to evaluate the PLES. In summary, the division of composite space is complicated, and some plots are difficult to merge into a certain type due to their multiple functions at the same time. Evaluation indexes selected by the index system method are mostly biased towards social and economic indicators, which is difficult to reflect the change and coupling coordination of the PLES truly.

### 2.2. The Spatial and Temporal Evolution of the PLES

Most studies on the spatiotemporal evolution of the PLES have been conducted with the help of the ArcGIS platform and combined with econometric models for quantitative analysis. In the literature, most of the existing studies analyzed the spatial evolution characteristics of PLES in terms of quantity, speed and direction of change, as well as pattern, equilibrium and patch change [27,31,32,33] based on the theoretical connotation and established the classification system of the PLES, through the land use dynamics, transfer matrix, Gini coefficient and landscape pattern analysis. The scale of the study is divided into national, regional, urban agglomeration, provincial, municipal and county areas. Since the natural environment and socioeconomic conditions of different regions differ greatly, the factors influencing the PLES are also different.

### 2.3. Study on the Conflict of the PLES

The study of “conflict” originated from sociology, and refers to the psychological or behavior contradictions caused by the incompatibility or opposition of different actors in goals [34,35,36]. With the increasing contradiction between economic and social development and resource and environmental protection, conflict analysis was introduced into land use and ecological protection, and the concepts of land conflict, land use conflict and space conflict were also put forwards. Among them, spatial conflict originates from the scarcity of spatial resources and spillover of spatial functions, and is a phenomenon of distribution opposition resulting from the competition of spatial resources in the process of man-land relationships [37,38]. Studies on spatial conflicts mainly focus on potential conflict identification, conflict level measurement, analysis of influencing factors, disclosure of evolutionary processes and coordinated governance [26,39,40,41]. The methods adopted include interview investigation methods [42], multicriteria decision analysis methods [43], pressure-state-response models [35] and adaptability evaluations [44]. Most studies believe that spatial conflict is the result of multiple factors [9,14]. In the early stage when human disturbance intensity is low, natural factors play a major role, while with the increase in population and the acceleration of urbanization and industrialization, human activities gradually play a leading role in shaping the landscape.

In summary, many scholars adopt a variety of methods to carry out research on territorial space or PLES, with diverse research perspectives and rich research results. However, due to the interdisciplinary nature and diversified research standpoint, a unified research route has yet to be formed. Prominent problems such as the contradiction between humans and land caused by the continuous improvement of the utilization intensity of space resources have become increasingly prominent. Research on the spatial conflict of the basin combined with land use conflict and PLES is still rare. The contributions of this paper mainly focus on the following aspects: First, land use conflicts and PLES are combined to reclassify land use types and construct the PLES classification system. Second, taking the YRB as the research object, the spatiotemporal evolution of production, living and ecology space is described, and a conflict model is established to analyze the regional differences in spatial conflicts. Finally, this study provides a scientific basis and decision-making reference for the coordinated and healthy development of PLES, and provides a theoretical basis, technical support and typical case analysis for the management and regulation of land use spatial conflicts in the basin.

## 3. Methods and Data

### 3.1. Research Area

**Figure 2:**
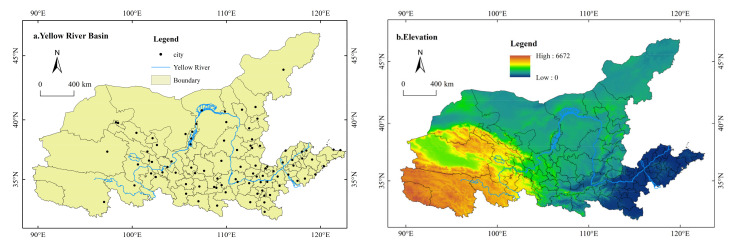
Administrative division of the Yellow River Basin (**a**) and elevation (**b**). Source: Developed by authors.

The Yellow River is located at 31°31′–43°31′ N, 89°19′–119°39′ E, starting from the Bayan Har Mountains on the Qinghai-Tibet Plateau and flowing eastward through nine provinces (regions): Qinghai, Sichuan, Gansu, Ningxia, Inner Mongolia, Shaanxi, Shanxi, Henan and Shandong (Figure 2). The total length of the Yellow River is 5464 km, and the total area of the basin is approximately 795,000 km^2^ (including an influx area of 42,000 km^2^). The Yellow River runs across the three strategic regions of East, Central and West China, and is a giant ecological corridor connecting the four geomorphic units of the Qinghai-Tibet Plateau, Inner Mongolia Plateau, Loess Plateau and North China Plain. The overall terrain of the basin is high in the west and low in the east. The average altitude of the western source region is over 4000 m, with numerous mountains and large topographic fluctuations. The elevation of the central region is 1000–2000 m; the geological structure is broken; and the soil texture is loose. Most of the eastern elevation does not exceed 50 m, and is mainly formed by the Yellow River sediment alluvial plain.

The YRB is a complex area of China’s “economy-society-environment” system. By the end of 2018, the total population of the YRB was 420 million, accounting for about 30% of the total population of China, and the regional GDP was 23.9 trillion yuan, accounting for more than a quarter of China. As the foundation and lifeblood of the development of northern China, it formed an obvious ladder development pattern of backwards upstream, rising midstream and developed downstream. In industrial and agricultural production, coal, oil, natural gas and mineral resources are abundant. Resource-based cities relying on the exploitation and processing of energy resources account for 47% of the total number of cities in the basin, and are important energy, chemical, raw materials and basic industrial bases. Agricultural and animal husbandry production also plays a pivotal role in the national economic pattern. The corresponding human living space is mainly concentrated in some river valleys in the upstream and plain areas in the midstream and downstream. Driven by natural environmental constraints and economic factors, the population distribution is dense in the middle and east, and sparse in the west. The ecological environment of the YRB is very fragile; the ecological functions of natural grasslands in the upper reaches are severely degraded; the middle reaches are faced with severe soil erosion problems; and urban life, industry and agriculture cause pollution to water resources. In recent decades, due to the rapid development of urbanization and industrialization, coupled with the complex geographical and climatic characteristics, the competition between production, living and ecology space has become an important factor restricting the sustainable development of this region.

### 3.2. Research Methods

#### 3.2.1. Construction of the PLES Classification System

**Figure 3:**
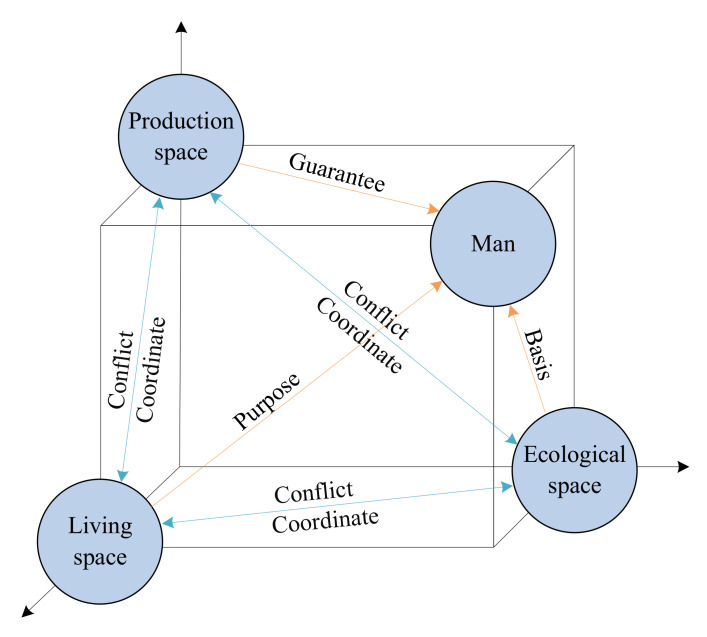
Conflict and coordination of PLES. Source: The authors.

PLES is the reflection of humans’ diversified demands for various products and services in daily work and life. It is the result of the interaction of different spatial environments and spatial scale elements, and is manifested in different utilization structures and ways in land use [2]. With the continuous deepening of research on PLES, many scholars divide land use into ecology space, production-ecology space, ecology-production space and living-production space from the perspective of the dominant and secondary functions of land use [9,25]. On the basis of exploring the connotation of PLES theory, some scholars established a spatial classification and evaluation system of PLES based on land use classification according to the national standard of land use classification [45]. Generally, a certain type of land space may have multiple functions at the same time. For example, cultivated land can not only be used for grain production, but also plays an important ecological role, but we usually think of arable land use primarily for food production. This paper draws lessons from the existing ideas and schemes of PLES spatial classification. According to the subjective intention of land use subjects and the dominant function of a certain type of land use, three spatial types of production, living and ecological are adopted to cover different land use patterns. The conflict and coordination among different land use patterns and the classification system are shown in Figure 3 and Table 1.

PS refers to the type of land use space that provides people with various products and services. This space takes land as the carrier to serve the most basic survival needs of human beings. It is the output of production and operation activities of the land use system, providing economic sources for most human beings and achieving the long-term goal of maintaining survival and development. [13]. Among them, dry land and paddy field are the main places for farmers to produce grain by providing agricultural products such as food and raw materials. Industrial, mining and transportation construction land mainly provides industrial products such as goods and service production, which is the main source of human mineral resources, and also includes transportation construction land serving transportation. Therefore, the production space is subdivided into APS and industrial production space (IPS).

LS refers to the type of land use space used by human beings for living, entertainment, science, education, culture and health, and some special purposes. This space aims to provide basic living conditions and security, and further meets the spiritual and cultural needs of human beings, which is the ultimate goal of the land use system [12]. As the current urban-rural dual structure is still relatively obvious, the living space is basically concentrated in urban and rural residential areas. Therefore, the living space is further subdivided into ULS and rural living space (RLS).

ES refers to the type of land use space that regulates the atmospheric environment, protects biodiversity, maintains soil and provides ecological products. This space is the foundation and support of production and living space, and is closely related to local natural resource endowment [9]. It can effectively promote regional sustainable development and ecological balance, and maintain the ecological stability of the natural environment. Therefore, the ecology space is further subdivided into forest ecology space (FES), grassland ecology space (GES), water ecology space and other ecology space (OES).

The essence of PLES evolution is a complex process of land use change. Under the influence of various driving factors such as the social economy and natural environment, it is manifested in the competition of various stakeholders for land use structure and form. In this dynamic process, PS guarantees various products and services produced and developed by human beings; LS meets the purpose of human habitation and social needs; and ES provides the basic material basis for human survival. Taking human beings as the center, various spatial types form the basic relationship of conflict and coordination with their unique functions. For example, ES meets people’s ecological needs and provides raw materials for PS. However, if human activities in PS and LS exceed the ecological carrying capacity, this will lead to environmental problems such as ecological pollution. Therefore, the evolution of PLES is a complex dynamic process, and its driving factors are mainly expressed in two aspects. In terms of social factors, the level of economic development, policies and regulations, residents’ life, population, industrial development, transportation and education all inevitably have an impact on the evolution of the PLES. Population factors, for example, population growth, distribution, age structure, comprehensive quality and migration will directly or indirectly promote the PLES evolution. In terms of natural factors, topography and climate characteristics are the basic factors affecting the distribution and change of PLES, such as slope, elevation and soil. In addition, the conversion of cropland to forest and grassland, land reclamation and deforestation can also affect the quantitative change of PLES.

#### 3.2.2. Land Use Transfer Matrix

The transformation of the PLES type and structure is realized by a land use transfer matrix model. The transfer matrix is a two-dimensional matrix that lists the transfer area of land use change according to the status quo of land cover in the same area and different phases, which serves as the basis of structural analysis and change direction analysis. It can not only reflect the area of each space type in a fixed region and at a fixed time statically, but also describe the area transfer of each space type at the beginning of the period and the area transfer of each space type at the end of the period [46,47]. This method is derived from the quantitative description of the system state and state transition in system analysis, and the formula is as follows:

(1) $$S_{ij}=\left|\begin{array}{c} S_{11}\;S_{12}\;\cdots \;S_{1n}\\ S_{21\;}\;S_{22}\;\cdots \;S_{2n}\\ \cdots \;\cdots \;\cdots \;\cdots \\ S_{n1}\;\;S_{n2}\;\cdots \;S_{nn} \end{array}\right|$$

where *S* is the land area; *n* is the total number of land types; and *i* and *j* are land use types at the beginning and end of the study period, respectively. In this paper, ArcGIS10.2 software is used to calculate the statistics of PLES space types in different periods, and then an Excel pivot table is used to establish the PLES transfer matrix.

#### 3.2.3. Spatial Conflict Index

Land use systems are complex, dynamic, and fragile, so spatial conflicts need to be considered from the three aspects of system complexity, stability and fragility [26]. To avoid excessive fragmentation of the spatial units in the study area, and taking into account factors such as the research scope, data type, spatial resolution, and patch conditions, under comprehensive testing and comparison, a 30 km × 30 km spatial grid was selected as the evaluation unit. The spatial plates that do not cover the entire grid in the boundary of the study area are calculated as a complete grid to calculate the landscape ecological index in each spatial unit to evaluate the degree of spatial conflict quantitatively. The calculation of SCI is [9]:

(2) SCI=CI+FI−SI

where SCI is the comprehensive index of spatial conflict, and CI, FI and SI are the spatial complexity index, vulnerability index and stability index, respectively.

The PLES complexity index reflects the gradual increase in the scale and intensity of land use due to rapid urbanization, and the continuous shaping of the surface morphology, resulting in the fragmentation of patches and the intensification of contradictions in space use. The area-weighted mean patch fractal dimension (AWMPFD) reflects the degree of interference of the domain plate on the measured patch, and to a certain extent reflects the impact of human activities on the spatial pattern. The higher the value is, the greater the external pressure on the patch. The AWMPFD in the landscape ecological index is used to characterize the spatial complexity index of PLES to measure the shape complexity of spatial patches. The formula is:

(3) $$\mathrm{AWMPFD}=\overset{m}{\underset{i=1}{\sum }}\overset{n}{\underset{j=1}{\sum }}\left[\frac{2\ln\left(0.25P_{ij}\right)}{\ln\left(a_{ij}\right)}\left(\frac{a_{ij}}{A}\right)\right]$$

where $P_{ij}$ is the perimeter of the patch; $a_{ij}$ is the area of the patch; A is the total area of the space types in the landscape; *i* and *j* are the *j*-th spatial types in the *i*-th spatial unit; m is the total number of spatial evaluation units in the YRB; and n is the total number of PLES types.

The PLES vulnerability index reflects the ability of space patches to withstand external pressure. At different stages, various land use types have different responses to external disturbances. The weaker the resistance is, the more vulnerable it is to external influences, and the higher the level of spatial conflict. Therefore, from the perspective of landscape ecology, using the vulnerability of various landscapes inside the space to calculate the PLES vulnerability index, the formula is as follows:

(4) $$\mathrm{FI}=\sum_{i=1}^{n}F_{i}\times \frac{a_{i}}{A}$$

where n is the total number of space types; $F_{i}$ is the vulnerability index of different space types, referring to existing research results [9,26]. Values are assigned to each space type: PS = 3, ES = 2, LS = 1; $a_{i}$ is the patch area; and A is the total area of the space type in the landscape.

The PLES stability index refers to the phenomenon where the regional spatial pattern fragments landscape patches under the interference of external pressure; the linear patches are “fishing nets”, and the dot-shaped patches show an agglomeration state, with increased density and separation, resulting in a decrease in the proportion of planar patches in the spatial unit. The more fragmented and complex the spatial form, the worse the stability within the spatial unit, the greater the spatial risk and the higher the intensity of spatial conflict. Therefore, the fragmentation degree of the landscape ecology is selected to represent the spatial stability index, and the formula is as follows:

(5) $$SI=1-PD\;PD=\frac{n_{i}}{A}$$

where SI represents the stability index of PLES. PD is patch density, and the larger the patch density is, the higher the degree of spatial fragmentation and the lower the stability of its spatial landscape; $n_{i}$ is the number of patches of the i-th space type in each space unit; A is the total area of the space type in the landscape.

Using the moving window method in Fragstats 4.2 software to measure the spatial conflict level of the PLES in the YRB, taking into account the characteristics of the research scale, data type, data volume and spatial patch conditions, comparing different size window units (including 1 km × 1 km, 4 km × 4 km, 7 km × 7 km, 10 km × 10 km) and referring to related research, it is found that the 4 km × 4 km window can better express the spatial conflict distribution characteristics. If the patch in the boundary area of the study area does not cover the entire unit area, the calculation is performed based on a complete unit area to calculate the index in each spatial unit mentioned above. Finally, the spatial conflict index is standardized to (0,1). According to the inverted “U”-shaped spatial conflict trajectory model, the space conflict index is divided into four categories: stable and controllable (0, 0.5) and basically controllable (0.5, 0.7), basically out of control (0.7, 0.9) and seriously out of control (0.9, 1).

### 3.3. Data

The PLES data of the YRB evolved from the land use type. These data come from the Resource and Environmental Science and Data Center of the Chinese Academy of Sciences. Among them, land use data include grid datasets of 1 km × 1 km in 1990, 2000, 2010 and 2020. The data are based on Landsat 8 remote sensing images, generated by the human-computer interaction visual interpretation and interpretation, including 6 primary types and 25 secondary types of cultivated land, forestland, grassland, water, residential land and unused land. Using ArcGIS 10.2 software, the land use types of the YRB were extracted based on the administrative division vector file; the PLES was divided by reclassification and other methods, and this software was used for mapping and data analysis.

## 4. Results and Discussion

### 4.1. Analysis of Evolution Characteristics of PLES

#### 4.1.1. Spatiotemporal Evolution Characteristics of PLES

With the help of ArcGIS 10.2 software, the area of the PLES of the YRB in 1990, 2000, 2010 and 2020 was extracted, and the changes in the spatial area of each category were calculated. The results are shown in Table 2. From 1990 to 2020, the PLES of the YRB was dominated by APS, FES, GES and OES. From 1990 to 2000, the overall ES showed a decreasing trend, with a decrease of 0.37%. The decrease in GES and OES was the most obvious. The expansion of PS and LS was 0.87% and 6.75%, respectively. Compared with other spaces, APS and ULS increased greatly. From 2000 to 2010, PS and ES decreased to varying degrees, among which APS and OES decreased significantly. In contrast, the LS expanded rapidly, with an increase of 16.25%, and the ULS expanded more rapidly than the RLS. From 2010 to 2020, with the continuous advancement of urbanization and industrialization, LS continued to expand, with an increase of 10.64%, and the IPS in the PS has an obvious increasing trend, while the ES continued to decrease, and the rate of decrease is 0.33%; thus, the reduction in GES and OES is the most obvious.

Overall, during the study period, the APS, GES and OES showed a shrinking trend, and the other four types of land use space had different degrees of expansion. Among them, ULS has the fastest growth rate, and the rest are IPS, RLS, WES and FES. The reason is that since the reform and opening up, the rich land and natural resources of the YRB gradually entered a stage of rapid development, coupled with its large population, rapid advancement of economic, social and cultural undertakings, rural surplus labor flows to cities and towns, urban land and transportation, and industrial and mining land rapidly increased, leading to the expansion of the ULS and IPS [48]. In addition, the increase in the population of rural Mesozoic families led to an increase in the demand for residential land. Coupled with the relaxation of rural land management and control, the phenomenon of “building new and not dismantling old” appeared in rural residential land, which in turn promoted the expansion of rural living space [49]. The increase in FES and WES was mainly due to the implementation of policies such as returning farmland to forests and ecological restoration. In contrast, while the economy and society are highly developed, a series of unreasonable economic behaviors, such as excessive grazing, reclamation and the mining of mineral resources continue to encroach on agricultural land and ecological land. Coupled with the extremely fragile ecological environment of the YRB and the impact of climate change [50], the GES and OES are gradually reduced.

**Figure 4:**
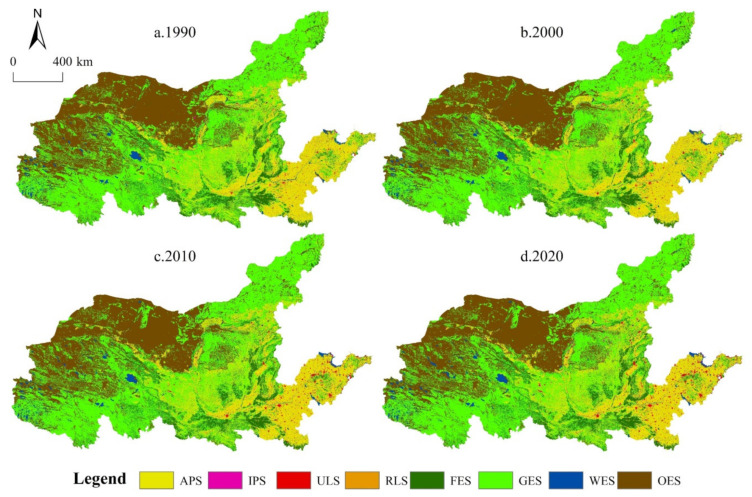
Spatial pattern characteristics of PLES in the YRB from 1990 to 2020: 1990 (**a**), 2000 (**b**), 2010 (**c**) and 2020 (**d**). Source: Developed by authors.

Based on the above analysis of temporal evolution characteristics, the spatial pattern characteristics of PLES in the YRB are further analyzed, as shown in Figure 4. From 1990 to 2020, the distribution pattern of PLES was basically consistent, and there was no significant indigenous change. The main characteristics are as follows: (1) The PS is dominated by APS, which is concentrated in traditional main grain producing areas such as Henan and Shandong downstream of the Yellow River. In addition, the Guanzhong Plain, Hetao Plain and Ningxia Plain are important agricultural production bases. The main reason is that these areas are suitable for agricultural production due to their superior human and natural environment, and strict farmland protection policies play a better role in protecting traditional agricultural areas. (2) The distribution of LS corresponds to the location of agricultural and industrial production space. The density of LS in the downstream area was significantly greater than that in the upstream and midstream areas, while the ULS showed a significant expansion trend, and the patch area increased significantly. The reason is that the vast midstream and upstream are mainly continental or plateau climates, with little precipitation and rugged terrain. The flat terrain of the downstream alluvial plain is more suitable for human habitation, and the excellent natural conditions breed the vast agricultural production areas. Due to the low traditional agricultural production technology, a large number of labors gather here to form dense rural settlements. In recent years, although the level of agricultural mechanization was greatly improved, and the demand for agricultural labor declined, due to the huge population base, rural settlements are still the main agricultural population. (3) The ecological spatial distributions are greater in the west and lower in the east. Among them, the FES is mainly distributed in southern Henan, Qinling Mountains and Taihang Mountains; the GES is widely distributed in arid and semiarid areas such as Qinghai, Gansu and eastern Inner Mongolia. Restricted by topography and climate differences, the WES and OES are mostly concentrated upstream of the Yellow River.

#### 4.1.2. The Structural Transformation Characteristics of PLES

To explore the internal transformation characteristics of the PLES in the YRB, based on the distribution map of the PLES in Figure 3, the spatial analysis function in ArcGIS is used to superimpose the distribution maps of the PLES in different periods to obtain the transfer model of PLES in the study area from 1990 to 2020 to clarify the direction and quantity of the conversion of land use types (Table 3 and Table 4). From 1990 to 2020, except for the reduction in APS, GES and OES, the structural space of other land types increased. Compared with the base period area, the IPS and UPS increased by a large margin, by 8828 km^2^ and 12,439 km^2^, respectively, during the period, and the growth rates were 225.22% and 169.52%. In internal conversion, from the perspective of roll-in structure, in terms of PS, APS is mainly converted from RLS and GES, and IPS is mainly converted from APS and GES; in terms of LS, both ULS and RLS are mainly converted from APS; in terms of ES, FES is mainly converted from APS and GES, and GES is mainly converted from APS and OES. The WES is mainly converted from APS, GES and OES, and OES is mainly converted from GES. From the perspective of roll-out structure, in terms of PS, APS is mainly transformed into ULS, RLS and GES, and IPS is most transferred to OES. In terms of LS, the transformation of ULS to other types of space is not obvious, and RLS is mainly turned to APS. In terms of ES, FES is mainly transformed into GES; GES is transformed into APS and OES; and OES is mainly transformed into GES.

**Figure 5:**
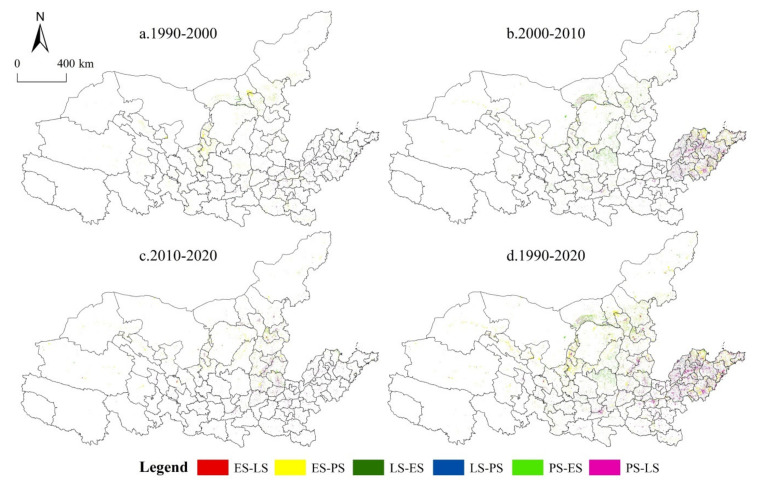
Spatial pattern evolution map of PLES in the YRB from 1990 to 2020: 1990 (**a**), 2000 (**b**), 2010 (**c**) and 2020 (**d**). Source: Developed by authors.

Based on the above analysis, the spatial distribution of land conversion in different periods is further discussed (Figure 5). Overall, the conversion areas of 1990–2000 and 2010–2020 were lower than those of 2000–2010. Specifically, from 1990 to 2000, the conversion of ES to PS was concentrated in central Inner Mongolia and Ningxia, and a small amount of PS to ES was also concentrated in central Inner Mongolia. The Henan and Shandong Province downstream of the YRB was dominated by PS to LS, and the distribution was more dispersed. From 2000 to 2010, the distributions of PS to ES and LS were the most extensive. Among them, PS to ES was distributed in the northwestern area of Shanxi, central Inner Mongolia and the Loess Plateau, and ES and PS to LS were mainly in the lower reaches of the YRB. At the same time, there was a small amount of ES to PS in the Shandong Peninsula and the Hexi Corridor of Gansu. From 2010 to 2020, the PS and ES transferred to LS, and were widely distributed in the whole basin, while the PS transferred to ES in the Shanxi Province and was the most prominent. In summary, from 1990 to 2020, the change of PLES in the YRB was dominated by the transformation between ES and PS, which was widely distributed upstream, midstream and downstream of the region. At the same time, the downstream region shows obvious characteristics of mutual transformation between PS and LS, and the transformation of ES and LS is sporadically distributed in various regions.

### 4.2. Analysis of Conflict and Change of PLES

#### 4.2.1. Time Evolution Characteristics

By calculating the spatial conflict index of four periods in the YRB, it is found that the average values of the spatial conflict index in 1990, 2000, 2010 and 2020 are 0.41, 0.43, 0.53 and 0.57, respectively (Table 5). With the rapid development of industrialization and urbanization, the intensity of spatial conflict in the YRB is on the rise, but still belongs to the basic controllable level. The proportion of space conflicts at the controllable level (stable controllable and basic controllable) remained between 74.17% and 81.70%, accounting for more than half of the total space units in the study area, and played an important role in controlling ecological risks and maintaining ecological security. The proportion of space units at stable and controllable levels continued to decline, falling by 17.23% in 2020 compared with 1990, while the proportion of space units at basic controllable levels increased by 31.34% at the end of the period compared with the beginning. The proportion of space units at the basic out of control conflict level showed a rapid growth trend and the largest increase, which increased by 46.29% in 2020 compared with 1990. The proportion of space units at the seriously out of control conflict level showed a wave-like rise, and decreased slightly from 1990 to 2000, but increased rapidly from 2000 to 2020, with an increase of 33.04%. In addition, the gap in the number of spatial units between the controllable level and the out of control level tended to narrow, and the gap between the basic controllable and basic out of control spatial units expanded rapidly. From the perspective of change trends, stable and controllable, basic controllable and basic out of control all showed linear changes, while the number of seriously out of control space units first decreased and then increased. Thus, with the gradual expansion of production and living space, out of control conflicts in some areas tend to expand, and the control over these regions should be strengthened to achieve the coordinated development of PLES in the YRB.

**Figure 6:**
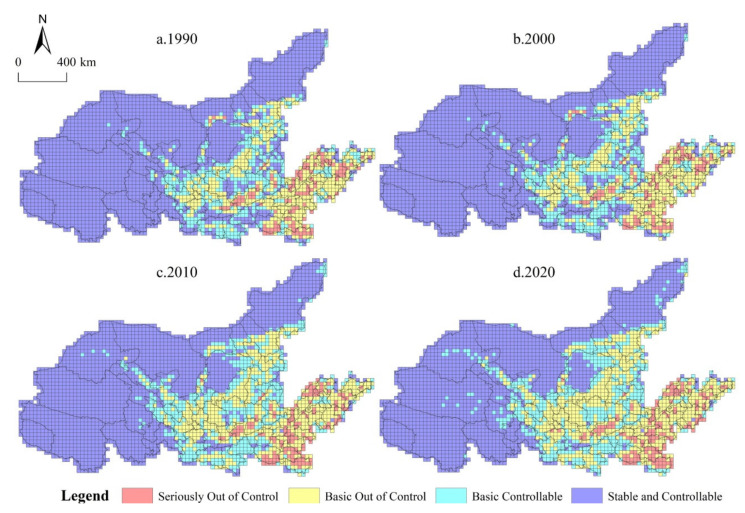
Changes in the PLES conflict index from 1990 to 2020: 1990 (**a**), 2000 (**b**), 2010 (**c**) and 2020 (**d**). Source: Developed by authors.

The spatial conflict index of each spatial unit was further calculated, and the spatial distribution of the conflict index of PLES in the YRB from 1990 to 2020 was visualized by using ArcGIS 10.2 software (Figure 6). In general, the distribution of spatial units with different conflict levels from 1990 to 2020 is relatively fragmented, except that the basic controllable types are concentrated; the basic controllable ones, the basic uncontrollable ones and the severe uncontrollable ones are all fragmented. In addition, the changes in spatial units with different conflict types during the study period show different characteristics. Longitudinally, the change degree of spatial units of different conflict levels was small before 2000, but the change after 2000 was characterized by fast speed and a wide range. Laterally, the stable and controllable areas were mainly concentrated upstream of the Yellow River, and the midstream and downstream were mainly out of control or seriously out of control. Specifically, stable and controllable spatial units are mainly distributed in the west and north of the YRB, and most are concentrated in Qinghai, Gansu and Inner Mongolia. The number of stable and controllable spatial units decreases significantly in the Hexi Corridor, southern Inner Mongolia, Shanxi and WeiHe River Basin. Basic controllable areas are mainly distributed in the midstream of the YRB, and the overall distribution of spatial units has a trend of transformation from fragmentation to centralized contiguous, which is mainly due to the transformation of stable controllable types to basic controllable, resulting in a basic controllable spatial unit distribution to expand outward. The basic out of control space units are widely distributed in the midstream and downstream parts of the Loess Plateau. With the passage of time, the basic out of control space in the Loess Plateau rapidly expands, while the basic out of control space in the downstream is stable and mainly distributed in the periphery of the seriously out of control space. The seriously out of control spatial units were scattered in the midstream and downstream of the YRB. The seriously out of control spatial units in the HeTao Plain changed to the basic out of control level from 2000 to 2020, and the seriously out of control spatial units in southern Henan and northern Shandong continued to spread, indicating that the PLES conflict downstream of the Yellow River increased.

#### 4.2.2. Conflict Differentiation Characteristics of PLES

**Figure 7:**
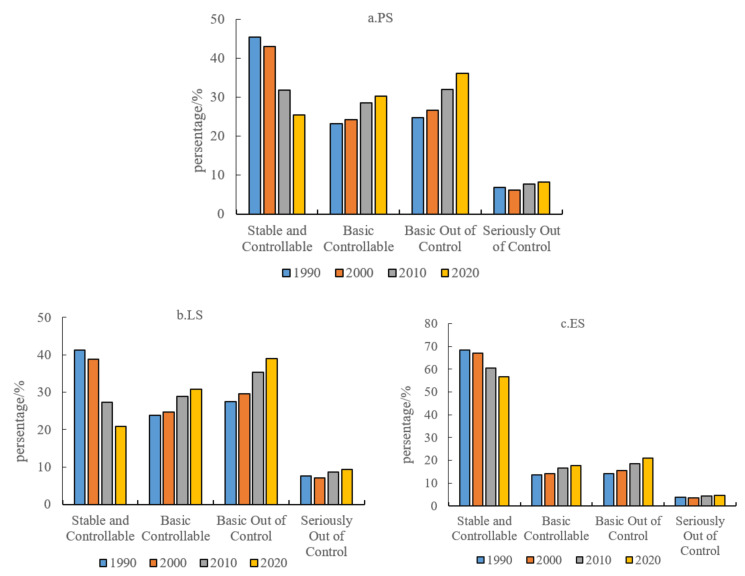
Spatial conflict levels of different spatial types in the YRB from 1990 to 2020: PS (**a**), LS (**b**), ES (**c**). Source: The authors.

The spatial conflict levels of the three spatial types in the YRB were calculated and statistically analyzed (Figure 7), and the results showed that there were differences in the composition of spatial conflict levels among different spatial types, and the level of out of control gradually increased, but all of them remained at the controllable level. The spatial conflict of PS is mainly controllable, accounting for more than 55%. The basic controllable conflict unit decreases year by year, while the stable controllable and basic out of control conflict unit increases year by year, and the serious out of control conflict unit shows a rising trend of fluctuation. PS is mainly concentrated in the midstream and downstream parts of the YRB. These areas have a high level of social and economic development, intensity of human development and construction activities, and the rapid development of urbanization and industrialization has led to prominent contradictions between people and land, increasing external pressure on space and increasing the intensity of space conflicts. The variation range of LS is similar to that of PS, and the spatial conflict is still controlled. The stable and controllable conflict units gradually decreased, with the largest decrease of 29.6% from 2000 to 2010. The basic controllable, basic out of control and serious out of control units increased by 29.3%, 42.4% and 22.5%, respectively. Compared with PS and LS, the variation range of conflict units in ES was smaller. Although stable controllable conflict units showed a slow downwards trend, controllable units remained above 70%. Basic controllable and basic out of control units showed a slight upwards trend, while seriously out of control units had the smallest variation range.

## 5. Conclusions and Suggestions

### 5.1. Conclusions

(1) From 1990 to 2020, PS and LS expanded by 0.87% and 6.75%, respectively, while ES decreased by 0.37%. In terms of spatial distribution, the APS of PS is concentrated in the traditional agricultural production area downstream, while the APS of the midstream and upstream is scattered. The distribution of LS corresponds to the agricultural and industrial production space. The density of LS in the downstream is significantly higher than that in the midstream and upstream, while the distribution of ecology space is more in the west and less in the east.
(2) APS, GES and OES decreased, and the rest of the space types showed a trend of expansion. IPS and ULS expanded rapidly. In terms of transition and transition structure, each space type has different transformation directions, but the transformation between ES and PS is the main transformation direction. In the downstream area, the transformation between PS and LS is mainly reflected in the mutual transformation of PS and LS, and the transformation distribution of ES and LS is scattered.
(3) From 1990 to 2020, the intensity of conflicts in PLES gradually increased, but it was still controllable. Stable and basic controllable space units accounted for more than 70%, and basic and seriously out of control space units gradually increased. In terms of space, the basic controllable space units are mainly distributed in the upstream region and relatively concentrated, while in the downstream region, they are mainly basic out of control and seriously out of control, and their distribution is relatively fragmented. Specifically, the variation range of conflict units in PS and LS is the same, while the variation range of conflict units in ES is smaller.

### 5.2. Suggestions

Based on the PLES perspective, this paper constructed a land use classification system in the YRB, and revealed the evolution characteristics and conflict index of PLES from 1990 to 2020. Strengthening spatial governance and improving land use efficiency is a complex system project that is affected by natural and human factors in various regions. Therefore, it is necessary to promote the scientific demarcation of the boundary of PLES in the YRB, strengthen the control and supervision of the boundary of various spatial types and divide the boundary of production, living and ecological spaces scientifically and reasonably according to the resource and environmental carrying capacity, spatial conflict level, spatial development suitability and social and economic development status of each region.

(1) Although most of the upstream areas are in a stable and controllable state, as China’s Water Tower and ecological spatial agglomeration area, ecological protection should be considered in the first place; the relationship between the production, living and ecological environment should be correctly handled, promote the construction of water conservation capacity, strengthen regional desertification control and strictly implement the principle of ecological access. The construction of nature reserves should promote ecological restoration, adhere to local conditions, comprehensively utilize engineering measures and biological measures to manage the ecosystem, control the ecology space at different levels and scales, delimit the rigid core elements and elastic space of ecological protection, reduce the frequency of human social and economic activities and alleviate the contradiction between human activities in the ecology space and the ecological environment.
(2) The midstream region should be committed to the spatial governance of soil erosion. On the one hand, it is important to protect forestland, grassland and unused land, enhance the soil and water conservation capacity, prevent the fragmentation of ecology space caused by urbanization and industrialization and take into account the economic and ecological benefits of land use. On the other hand, ecological restoration should be actively promoted to improve the management efficiency in the middle reaches. For irrigated agricultural areas, excessive consumption of water resources in agriculture and industry should be prevented; the proportion of ecological water should be increased; the intensity of land development should be strictly controlled; industries related to ecological environment protection should be developed; and the coupling and coordination level of PLES should be improved.
(3) The downstream region, as the region with the most serious conflict, is densely populated and has high industrial and agricultural production intensity. First, it is necessary to improve the quality of the population while reasonably controlling the size of the population, with land efficient and intensive use as the development goal, optimize the industrial layout, focus on cultivating new industrial systems, strictly control the disorderly expansion of urban and rural construction land, improve the utilization efficiency of existing construction land and alleviate the contradiction between industrial and agricultural production and ecological protection to create a green ecological corridor in the lower reaches of the Yellow River. Second, for the vast agricultural production space, the level of agricultural mechanization should be improved, protect high-quality cultivated land resources, improve land output income and reduce dependence on land resources. Finally, the concept of green development was combined with technical means, using the theory and technology of sponge cities, green buildings and ecological materials to promote the construction of urban ecological civilizations and strengthen the positive impact of land invisible forms on the ecological environment.

## Figures and Tables

**Table 1:** Classification system of PLES.

First-Level Classification	Second-Level Classification	Third-Level Classification
Production space	Agricultural production space	Paddy field, Dry land
	Industrial production space	Industrial, mining and transportation construction land
Living space	Urban living space	Urban land
	Rural living space	Rural residential area
Ecology space	Forestland ecology space	Forestland, shrubbery forest, Sparse woodland, Other woodlands
	Grassland ecology space	High, Medium and Low coverage grassland
	Water ecology space	Canal, Lakes, Reservoir pit, Permanent glacier snow, tidal flat
	Other ecology spaces	Sand land, Gobi, Saline-alkali land, Everglade, Bare land, Bare rock texture, Other

Source: The authors.

**Table 2:** The area and changes in the PLES of the YRB from 1990 to 2020 (km^2^).

Year	PS		LS		ES			
	APS	IPS	ULS	RLS	FES	GES	WES	OES
1990	475,388	3926	7356	43,377	209,919	1,043,018	56,220	710,624
2000	479,304	4166	9544	44,613	209,381	1,035,888	57,123	709,888
2010	469,323	5440	16,226	46,734	211,935	1,039,876	58,748	701,656
2020	461,927	12,768	19,826	49,835	212,412	1,035,644	62,179	695,437
Change between 1990 and 2000	3916	240	2188	1236	−538	−7130	903	−736
Change between 2000 and 2010	−9981	1274	6682	2121	2554	3988	1625	−8232
Change between 2010 and 2020	−7396	7328	3600	3101	477	−4232	3431	−6219
Change between 1990 and 2020	−13,461	8842	12,470	6458	2493	−7374	5959	−15,187

Source: The authors.

**Table 3:** The transition matrix of the spatial structure of the PLES from 1990 to 2020 (km^2^).

1990	2020								Roll-Out
	APS	IPS	ULS	RLS	FES	GES	WES	OES	
APS	-	4104	9509	10,647	3826	10,929	2432	1363	42,810
IPS	157	-	188	103	39	330	268	402	1487
ULS	238	14	-	214	22	70	6	5	569
RLS	4118	171	1944	-	57	208	69	40	6607
FES	2476	496	245	292	-	3286	205	305	7305
GES	16,653	3473	665	1400	5031	-	2821	17,407	47,450
WES	1998	434	300	139	116	832	-	1537	5356
OES	3704	1623	157	270	705	24,420	5367	-	36,246
Roll-in	29,344	10,315	13,008	13,065	9796	40,075	11,168	21,059	-

Source: The authors.

**Table 4:** Statistics on the changes and characteristics of the PLES utilization from 1990 to 2020.

Type	Area/km^2^					
	Roll-In Rate	Roll-Out Rate	The Total Variation	Exchange Variation	Net Increase	Rate of Increase and Decrease
APS	6.35%	9.01%	72,154	58,688	−13,466	−2.83%
IPS	80.88%	37.88%	11,802	2974	8828	225.22%
ULS	65.71%	7.74%	13,577	1138	12,439	169.52%
RLS	26.22%	15.23%	19,672	13,214	6458	14.89%
FES	4.61%	3.48%	17,101	14,610	2491	1.19%
GES	3.87%	4.55%	87,525	80,150	−7375	−0.71%
WES	18.01%	9.53%	16,524	10,712	5812	10.60%
OES	3.03%	5.10%	57,305	42,118	−15,187	−2.14%

Source: The authors.

**Table 5:** Statistics of the PLES Conflict Index in the YRB from 1990 to 2020.

Level of Conflict	Number of Space Units				Percentage (%)			
	1990	2000	2010	2020	1990	2000	2010	2020
Stable and Controllable	2112	2069	1866	1748	68.2	66.81	60.25	56.44
Basic Controllable	418	436	516	549	13.5	14.08	16.66	17.73
Basic Out of Control	445	480	577	651	14.37	15.5	18.63	21.02
Seriously Out of Control	122	112	138	149	3.94	3.62	4.46	4.81
Average	0.41	0.43	0.53	0.57	-	-	-	-
Total	3097	3097	3097	3097	100	100	100	100

Source: The authors.

## Data Availability

The data presented in this study are openly available in the Resource and Environmental Science Data Center of the Chinese Academy of Sciences.

## Appendix A

**Table A1:** Abbreviation table.

Full Name	Abbreviation	Full Name	Abbreviation
Production-living-ecology space	PLES	Rural living space	RLS
Yellow River Basin	YRB	Forestland ecology space	FES
production space	PS	Grassland ecology space	GES
living space	LS	Water ecology space	WES
ecology space	ES	Other ecology spaces	OES
Agricultural production space	APS	Spatial conflict index	SCI
Industrial production space	IPS	area-weighted mean patch fractal dimension	AWMPFD
Urban living space	ULS	patch density	PD

Source: The authors.
